# Sarcoidosis Presenting as a Right Paraesophageal Mass Abutting the Left Atrium

**DOI:** 10.7759/cureus.5403

**Published:** 2019-08-16

**Authors:** Julien Exposito, Jason R Mammino, Timothy J O'Toole, Furqan Haq, Said Awad

**Affiliations:** 1 Internal Medicine, Largo Medical Center, Largo, USA; 2 Internal Medicine, Nova Southeastern University Dr. Kiran C. Patel College of Osteopathic Medicine, Fort Lauderdale, USA; 3 Internal Medicine, Oak Hill Hospital, Tampa, USA

**Keywords:** sarcoidosis, middle mediastinum, pericardial mass, peribronchial mass, incidental findings

## Abstract

We herein report a unique case of sarcoidosis in a 44-year-old Caucasian female. The patient was initially evaluated for hematochezia and diagnosed with diverticulitis after computed tomography (CT) scan of the abdomen and pelvis. This imaging also incidentally showed a large mass abutting the esophagus. Further imaging of the mass revealed widespread lymphadenopathy in the thorax without any respiratory or classic B symptoms present. The remaining workup caused concern for possible lung or hematologic malignancy. Following biopsy of a thoracic lymph node, the patient was diagnosed with sarcoidosis. This patient met only one of the usual demographic criteria associated with sarcoidosis and none of the lab criteria. While sarcoidosis is typically a benign disease, this case exemplifies how it may appear as a more sinister entity and warrants extensive workup to rule out malignancy.

## Introduction

Lymphadenopathy attributed to lymphoma, sarcoidosis, or metastatic lung cancer is the most common lesion to be found in the middle mediastinum. The highest incidence of sarcoidosis occurs in African Americans women between the ages of 30 and 39. For Caucasians, the incidence peaks from 40 to 49 years of age in both males and females [1]. The highest annual incidence is observed in European Americans at 3 to 10 cases per 100,000 people per year [2]. African Americans are three times more likely to develop sarcoidosis compared to Caucasian Americans [1]. Benign cystic tumors in the tracheobronchial tree and pericardium can also develop in this location. The location of the mass determines subsequent testing with the goal of tissue biopsy for definitive diagnosis. Patients diagnosed with lymphoma usually present with classic B symptoms such as fevers, weight loss, or night sweats. Other symptoms due to mass effect may include chest pain, dyspnea, wheezing, stridor, hoarseness, dysphagia, or eventual superior vena cava syndrome with heart failure sequelae due to compression of the mediastinal structures. Mediastinal masses are typically found when they produce mass effect or seen incidentally on imaging.

## Case presentation

**Figure 1:**
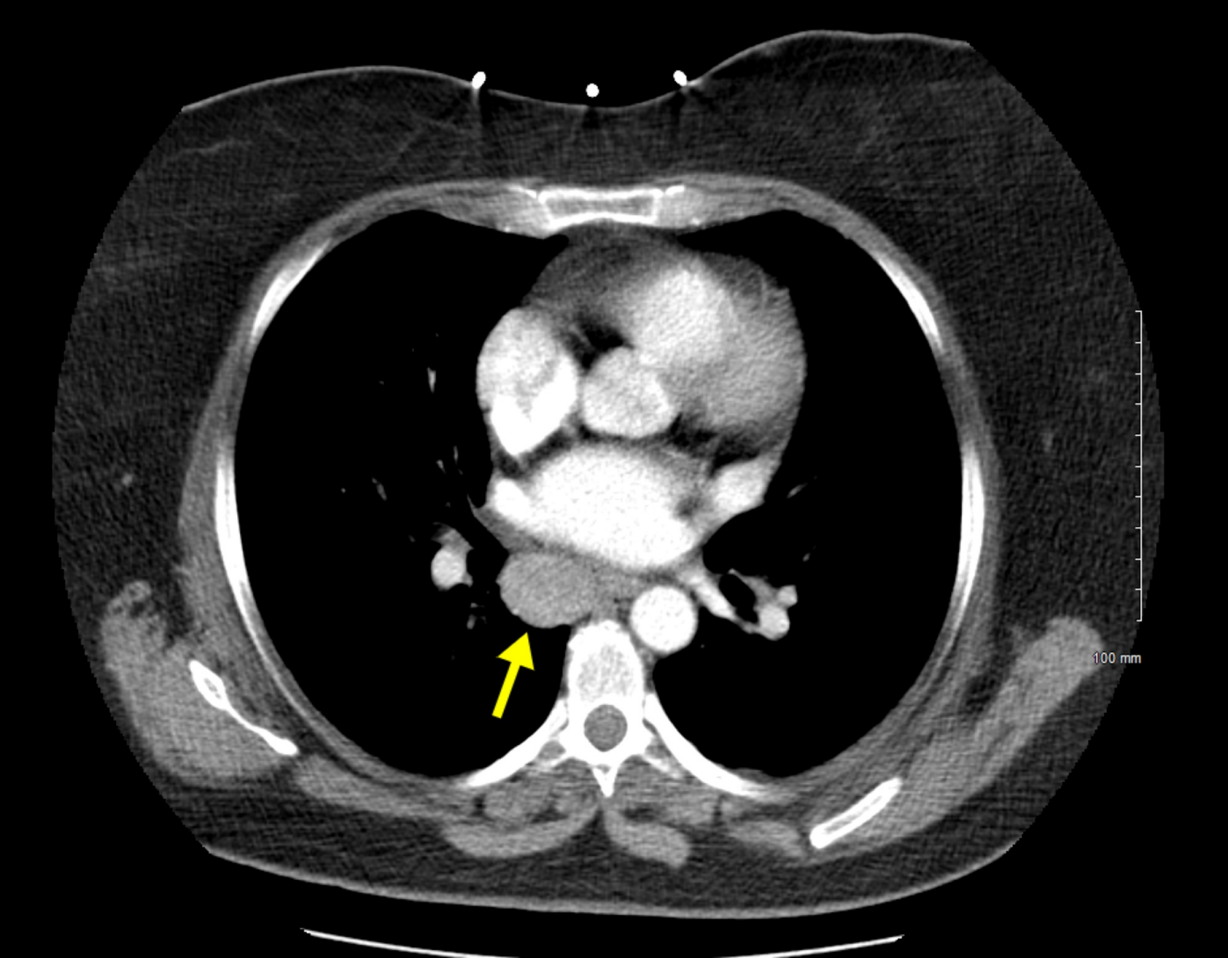
Computed tomography (CT) of the abdomen and pelvis 2.4 cm x 2.0 cm soft tissue mass in the right paraesophageal location abutting the esophagus as well as the left atrium in the lower chest region.

A 44-year-old Caucasian female with a significant past medical history of diverticulitis, hypertension, obesity, and tobacco use presented for evaluation of hematochezia and left lower quadrant abdominal pain for the past eight hours. A computed tomography (CT) of the abdomen and pelvis was obtained which showed acute diverticulitis along with an incidental right paraesophageal mass abutting the left atrium which was new from prior studies (Figure 1). 

**Figure 2:**
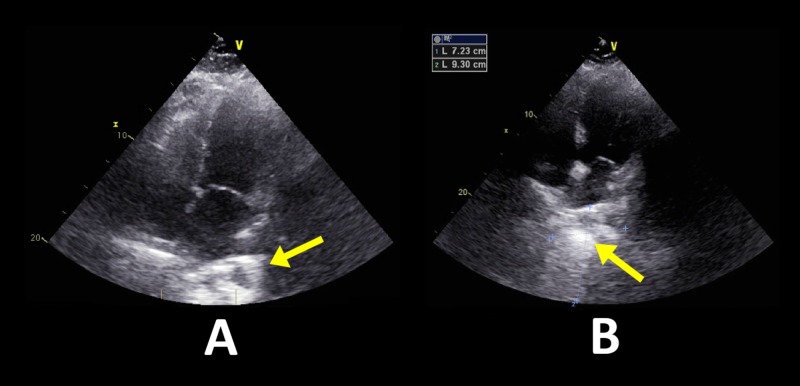
Transthoracic echocardiogram Left ventricular ejection fraction 65% with grade 1 diastolic dysfunction. A: Four chamber view at the end of diastole exhibiting no signs of encroachment upon the left atrium; B: Four chamber view at the end of systole better visualizing an echogenic mass just anterior to the left atrium.

A transthoracic echocardiogram (TTE) was ordered to evaluate for possible cardiac compromise from the mass encroaching on the left atrium. The TTE showed a normal left ventricular ejection fraction of >65% with no compromise in the left atrial filling (Figures 2A-2B). 

**Figure 3:**
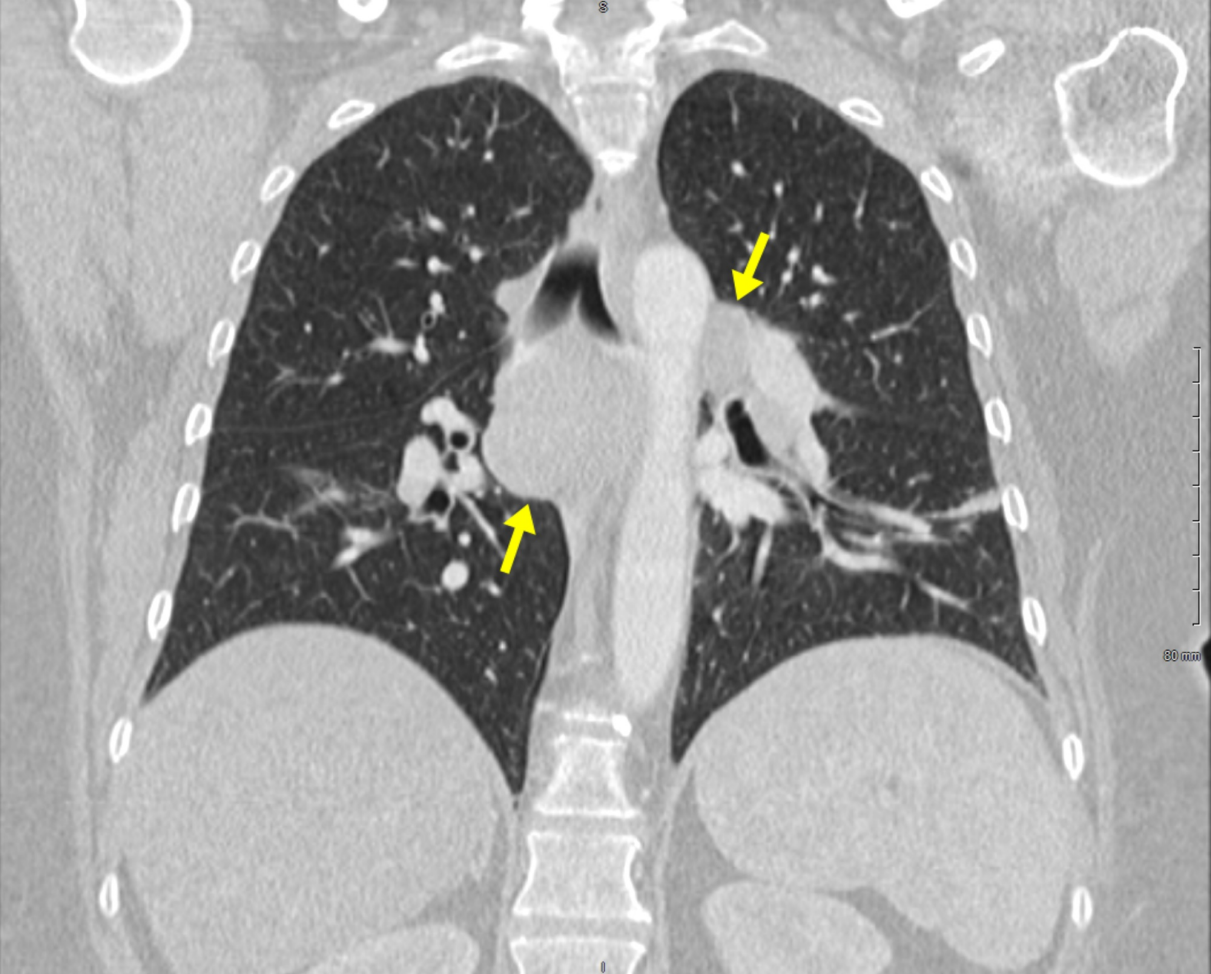
Computed tomography (CT) of the chest Numerous enlarged lymph nodes can be seen within the mediastinum. The largest is in the sub-carinal area measuring 3.1 cm x 4.0 cm. The second largest is in the right peritracheal area measuring 2.1 cm x 2.6 cm. Enlarged lymph nodes are also seen within each hilum, left greater than right.

A follow-up CT of the chest with contrast showed widespread mediastinal lymphadenopathy and bilateral hilar adenopathy concerning for malignancy, lymphoma, or sarcoidosis (Figure 3). 

At this time a pulmonologist was consulted who recommended an excisional lymph node biopsy. CT surgery was consulted and performed the excisional biopsy via mediastinoscopy. The pathology exhibited effacement of the nodal architecture by multiple small granulomas with minimal necrosis or caseation indicative of sarcoidosis. Acid-fast bacillus and periodic acid-Schiff sections were negative for organisms and no polarizable foreign material was visualized. Hematology/Oncology was consulted and they recommended an outpatient positron emission tomography (PET) scan and bone marrow biopsy to evaluate for metastasis. The patient’s beta 2- microglobulin were elevated suggesting possible lymphoma. The patient denies weight loss, fevers, night sweats, chills, cough, chest pain, and shortness of breath. Her hematochezia resolved during the first day of her hospitalization. An esophagogastroduodenoscopy was performed which did not show any signs of active bleeding. Due to her acute diverticulitis, it was recommended that this patient should follow up with outpatient gastroenterology for colonoscopic evaluation in 4-6 weeks.

## Discussion

This case illustrates how incidental findings are important to evaluate especially if there is concern for malignancy or compromised cardiac function. This patient was completely asymptomatic, not showing any of the typical B symptoms attributed to lymphoma. Evaluating the cardiac function of the patient with TTE was also vitally important as this mass was abutting the left atrium. If this mass had continued to grow, it could have eventually compromised left atrial filling leading to pulmonary edema and left-sided heart failure. Similar cases have been reported in the literature with initial concerns for malignancy later proving to be sarcoid in origin.

One such case of a 65-year-old female presenting with dyspnea, fatigue, and weight loss was evaluated and determined to have probable lung malignancy based on lab work, imaging, and endoscopic evaluation [3]. It was only after biopsy and eventual lymph node resection that lung malignancy was ruled out and sarcoid was determined. Comparable to our case is that the imaging was highly suspicious for lymphoma. The diagnosis was further supported by incidental lab findings which revealed elevated beta 2-microglobulin. What makes our case unique is that all of these findings were totally incidental and not in any way related to her initial complaint. Our patient also did not fit the typical demographics of a patient with sarcoidosis, which tend to affect African American women in their 20s-30s. Our patient also had none of the typical signs and symptoms of sarcoid such as cough, dyspnea, and fatigue. These along with normal angiotensin‐converting enzyme (ACE) levels and normal calcium level kept the diagnosis of sarcoidosis low on the differential.

Another case that further demonstrates how our case does not meet any of the usual sarcoidosis demographic and lab criteria is of a 53-year-old female of African-Caribbean descent who presented with a chief complaint of vomiting and constipation with additional low mood and weight loss [4]. Initial labs showed markedly elevated calcium along with elevated erythrocyte sedimentation, C-reactive protein, and serum protein. The initial concern in this patient was myeloma with a secondary hypercalcemia which was subsequently ruled-out after serum protein electrophoresis showed a polyclonal proliferation and urine protein electrophoresis without Bence-Jones protein. Only after this was sarcoidosis considered and the patient was found to have a markedly elevated ACE level. Her initial complaints were determined to be resulting from the hypercalcemia and resolved with intravenous hydration. The sarcoidosis was treated with steroids. This case study meets many of the typical criteria of sarcoidosis which include female gender, African descent, hypercalcemia, and elevated ACE. This is in contrast to our patient who is Caucasian and whose labs revealed normal calcium and ACE levels. This case was similar to ours due to the initial concern and eventual ruling out of malignancy as the root cause of the patient’s presentation.

## Conclusions

This case illustrates the importance of thoroughly evaluating incidental findings. This patient had an atypical presentation of sarcoidosis that was only identified by imaging that was done for a different complaint that the patient was presenting with. By further evaluating these incidental findings, we were able to make the diagnosis of sarcoidosis. The patient did not present with the typical symptoms of sarcoidosis making the case that much more challenging in identifying the cause of widespread mediastinal lymphadenopathy as well as the mass that was abutting her left atrium. 
